# Next-Generation Sequencing for Minimal Residual Disease Detection in Pediatric Acute Lymphoblastic Leukemia: Technological Advances, Clinical Translations and Current Challenges

**DOI:** 10.3390/cimb48050512

**Published:** 2026-05-15

**Authors:** Nan Liu, Yi Zheng, Xiaojun Xu

**Affiliations:** Department of Hematology-Oncology, Children’s Hospital of Zhejiang University School of Medicine, National Clinical Research Center for Children and Adolescents’ Health and Diseases, Hangzhou 310052, China; 6514032@zju.edu.cn (N.L.); 22318424@zju.edu.cn (Y.Z.)

**Keywords:** next-generation sequencing, minimal residual disease, acute lymphoblastic leukemia, pediatric

## Abstract

Minimal residual disease (MRD) is the most robust independent prognostic biomarker for pediatric acute lymphoblastic leukemia (ALL). Conventional MRD detection assays suffer from insufficient sensitivity and inherent technical limitations, failing to identify ultra-low-level leukemic blasts and thereby contributing to disease relapse. Next-generation sequencing (NGS)-based MRD detection (NGS-MRD) overcomes these drawbacks by targeting immunoglobulin (Ig)/T-cell receptor (TCR) gene rearrangements and enabling the precise quantification of residual leukemic clones. In recent years, NGS-MRD has undergone extensive technological optimization in target panel design, result interpretation and sample type expansion, and has been validated for its clinical utility in therapeutic threshold definition, prognostic stratification, post-therapy monitoring and treatment adjustments in pediatric ALL. This review synthesizes the latest technological refinements and clinical applications of NGS-MRD in pediatric ALL, critically discusses the current challenges that limit its routine clinical use, and proposes future research directions to address these issues.

## 1. Introduction

Acute lymphoblastic leukemia (ALL) accounts for the majority of childhood hematological malignancies, with a long-term overall survival (OS) rate of approximately 90% in the modern era of risk-adapted chemotherapy, improved supportive care and standardized prognostic stratification [1,2,3]. Despite this remarkable progress, 10–20% of pediatric patients experience disease relapse, with a 5-year OS rate of only 50% post-relapse [3,4,5]. Moreover, long-term survivors face severe treatment-related late complications, such as neurotoxicity, growth retardation, secondary malignant neoplasms and reproductive function damage [6,7,8]. The core challenge in contemporary pediatric ALL management is to balance curative efficacy with minimal treatment toxicity via precise, personalized therapeutic strategies—an objective that hinges on the accurate assessment of minimal residual disease (MRD).

MRD, defined as the presence of submicroscopic leukemic blasts in the bone marrow or peripheral blood after treatment, is the gold standard for evaluating treatment response and predicting relapse risk in pediatric ALL. Multiparameter flow cytometry (MFC) and polymerase chain reaction (PCR) are the clinical standards for MRD detection. MFC quantifies MRD based on the specific surface immunophenotypes of leukemic cells, whereas PCR identifies MRD by targeting leukemia-specific fusion gene transcripts or patient-specific molecular markers of immunoglobulin/T-cell receptor (Ig/TCR) gene rearrangements. Although both techniques achieve a sensitivity of 10^−4^, they harbor distinct molecular and technical limitations. MFC relies on viable cells, leading to poor stability of sample cell suspensions, and is susceptible to drug-induced antigenic drift of leukemic blasts [9,10], including downregulation of markers such as CD19 induced by blinatumomab and chimeric antigen receptor T (CAR-T) therapy [11]. PCR requires personalized primer design for individual leukemic clones, carries the risk of false negatives due to oligoclonal or subclonal rearrangement alterations, and may produce false positives from non-specific primer annealing. Furthermore, samples with extremely low tumor burden often yield positive but non-quantifiable MRD signals [12].

Next-generation sequencing (NGS) has emerged as a transformative technology for MRD detection, by amplifying Ig/TCR gene rearrangements (clonal molecular signatures of leukemic lymphoblasts) using universal primers with molecular barcoding. This approach eliminates the need for personalized primer design and achieves a sensitivity of 10^−4^ to 10^−7^, significantly outperforming conventional assays [13,14]. However, it is important to distinguish this technical metrology parameter from clinically actionable MRD thresholds, which are typically set at 10^−6^ for treatment decisions and risk stratification. Clinical studies on NGS-detected ultra-low MRD (10^−6^–10^−7^) are currently scarce in pediatric ALL. Table 1 summarizes the comparison of various MRD detection methods.

The clinical translation of NGS-MRD was landmarked by the US Food and Drug Administration (FDA) approval of ClonoSeq for ALL in 2018 [15], followed by the publication of a standardized NGS-MRD workflow by the EuroClonality-NGS consortium in 2019 [16], which established a framework for the uniform clinical application of this technology. Since then, numerous clinical studies have validated the superior value of NGS-MRD in pediatric ALL, and it has been incorporated into Chinese consensus and guidelines for pediatric leukemia MRD detection [17,18].

This review focuses on the technological optimization of NGS-MRD for pediatric ALL, the clinical translational evidence for its utility in prognostic stratification and post-therapy monitoring, directing personalized therapeutic interventions, and the unresolved current challenges in its molecular characterization and widespread clinical implementation. We also outline key future research directions to advance the personalized management of pediatric ALL via NGS-MRD, aligning with the need to address critical molecular and clinical gaps in the field.

## 2. Technological Optimization of NGS-MRD for Pediatric ALL

The clinical utility of NGS-MRD is predicated on rigorous technological optimization to ensure high sensitivity, specificity and reproducibility. Key advances in recent years have focused on the precision design of molecular target panels, standardized interpretation of sequencing reports, and the expansion of non-invasive sample types—all of which address inherent limitations of early NGS-MRD assays and enhance its clinical applicability.

### 2.1. Precision Design of Ig/TCR Gene Target Panels

NGS-MRD targets clonal Ig/TCR gene rearrangements and the unique molecular fingerprints of leukemic B/T cells, and the design of the target panels is tailored to the distinct molecular features of B-ALL and T-ALL.

For B-ALL, the immunoglobulin heavy chain (IGH) gene rearrangement is the primary molecular target, with a single detection rate of 87.7%, whereas immunoglobulin kappa (IGK) and lambda (IGL) were found only in approximately half of the patients. IGH-MRD retains independent prognostic value in B-ALL, making it the core target for clinical NGS-MRD panels, but the prognostic significance of IGK/IGL positivity is compromised by non-specific clonal rearrangements in healthy individuals [19]. Our recent work confirmed that IGK and IGKDE rearrangements are common in normal individuals, and that non-specific IGK clones consistently exhibit an N-region length ≤ 4 nucleotides at the V-J segments. Accordingly, molecular filtering of these non-specific clones based on the N-region length and their distribution probability in healthy cohorts markedly improves the reliability of IGK rearrangement for MRD monitoring [20]. However, it is important to critically acknowledge that this conclusion is derived from a single-center retrospective cohort; large-scale multicenter prospective validations are warranted to establish universally applicable filtering algorithms before adopting them in routine diagnostic protocols.

For T-ALL, TCR gene rearrangements exhibit more complex molecular patterns, with high structural heterogeneity, deletion rates and significant interference from clonally diverse normal T cells, posing major challenges for NGS-MRD design. In early T-cell precursor ALL (ETP-ALL), a high-risk T-ALL subtype, 63% of patients have detectable TCR delta (TCRD) rearrangements. These rearrangements arise earlier in T-cell development and display greater molecular stability, thereby positioning TCRD as a key target for identifying early residual leukemic clones [21]. In other T-ALL subtypes, clonal TCR beta (TCRB) sequences were detected in 68.5% of T-ALL pretreatment samples; combining TCRB and TCR gamma (TCRG) increased the overall detectability to 82.4%. Moreover, NGS-MRD positivity showed no significant correlation with disease-free survival at either the end of induction (EOI) or the end of consolidation (EOC) [22]. However, this retrospective finding should be interpreted cautiously. The lack of prognostic correlation may stem from technical limitations rather than true clinical irrelevance. To date, the clinical approval of NGS-MRD for T-ALL has been impeded by the intricate molecular landscape of TCR rearrangements, largely due to unresolved challenges in ensuring consistent sensitivity and specificity.

### 2.2. Standardized Interpretation of NGS-MRD Reports

**Figure 1:**
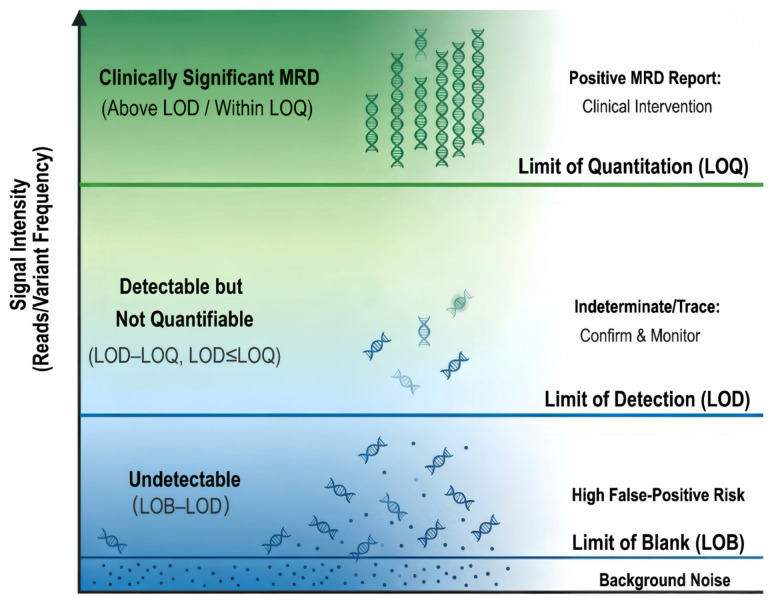
NGS-MRD molecular thresholds: LOB, LOD, and LOQ interpretation.

Accurate clinical interpretation of NGS-MRD data requires a rigorous methodological validation system based on three core quantitative molecular indicators, the limit of blank (LOB), limit of detection (LOD) and limit of quantitation (LOQ), as shown in Figure 1. These indicators collectively guide clinical decision-making by defining the authenticity and quantifiability of clonal Ig/TCR signals, and have replaced the simplistic binary “positive/negative” model of conventional MRD assays [23,24]:

• LOB: The background molecular threshold for distinguishing true clonal signals from sequencing noise; signals below LOB are defined as true negative.
• LOD: The key sensitivity parameter, representing the lowest level of MRD that can be reliably detected; signals above LOD but below LOQ are detectable but not accurately quantifiable, requiring further molecular characterization to distinguish false positives from potential low-level true positives.
• LOQ: The minimum threshold for precise quantitative analysis of clonal signals; signals above LOD and quantifiable within the LOQ range indicate clinically significant MRD and are a core biomarker of poor prognosis.

This standardized molecular interpretation framework ensures the reproducibility of NGS-MRD results across different laboratories, a critical prerequisite for its clinical translation and standardization.

### 2.3. Expansion of Non-Invasive Sample Types for Molecular Monitoring

Bone marrow (BM) is the gold standard sample for MRD detection, but the expansion of NGS-MRD to peripheral blood (PB) has enabled non-invasive, dynamic molecular monitoring of pediatric ALL—an important advance for clinical practice.

Although PB NGS-MRD signals are 1–2 log orders lower than those in BM [25,26,27], PB NGS-MRD still exhibits significantly higher sensitivity than BM MFC-MRD. In the absence of a predefined detection threshold, PB NGS-MRD identified an additional 13% of MRD-positive pediatric ALL cases (10/77) that were undetectable with conventional assays, all of which had clear clinical implications: five cases developed BM/extramedullary relapse, four cases underwent HSCT and one case was lost to follow-up [27]. Considering the limitations of its small retrospective design and potential selection bias, large prospective multicenter trials are required to validate the non-inferiority of PB to BM. These findings confirm that PB is a valid non-invasive alternative to BM for NGS-MRD monitoring, and its ease of collection enables serial sampling to capture dynamic changes in clonal leukemic burden, improving the timeliness of relapse prediction.

The clinical validity of PB NGS-MRD has also been verified in adult ALL, with a strong Pearson correlation coefficient (0.87, *p* < 0.001) between PB and BM results, and a sensitivity of 87% and specificity of 90%. Notably, PB NGS-MRD detects MRD 100% in advance (median 90 days) in adult ALL patients with post-HSCT relapse, and provides early relapse warning in 85% of patients with post-CAR-T relapse (median 60 days in advance) [28]. This cross-age validation highlights the broad applicability of PB NGS-MRD for non-invasive molecular monitoring of ALL.

### 2.4. Expansion of Liquid Biopsies: Application of Cell-Free DNA (cfDNA) and Cerebrospinal Fluid (CSF) in Extramedullary and Central Nervous System Monitoring

In addition to PB and BM, groundbreaking progress has been made in non-invasive or minimally invasive liquid biopsies over the past few years, successfully expanding the application of NGS-MRD to cfDNA and CSF. Central nervous system (CNS) relapse remains a major cause of treatment failure and mortality in pediatric ALL. Conventional CSF cytology and MFC often yield false-negative results due to insufficient sensitivity or the high fragility of leukemic cells in vitro when the leukemic burden is extremely low [29]. A recent study has demonstrated that targeted amplification or nanopore sequencing of cfDNA from CSF or plasma can effectively overcome the limitations of conventional assays that rely on intact tumor cells. Given its extremely short half-life (15–120 min), tumor-derived cfDNA offers a better real-time snapshot of dynamic tumor burden than cellular assays. Even when conventional MFC fails to detect intact blasts in PB or BM, cfDNA-MRD can sensitively capture dynamic changes in minimal leukemic clones, providing an earlier warning for extramedullary and CNS relapses [30]. This underscores the tremendous potential of incorporating cfDNA and CSF into routine NGS-MRD monitoring systems to guide CNS-directed interventions.

## 3. Clinical Translational Applications of NGS-MRD in Pediatric ALL

**Figure 2:**
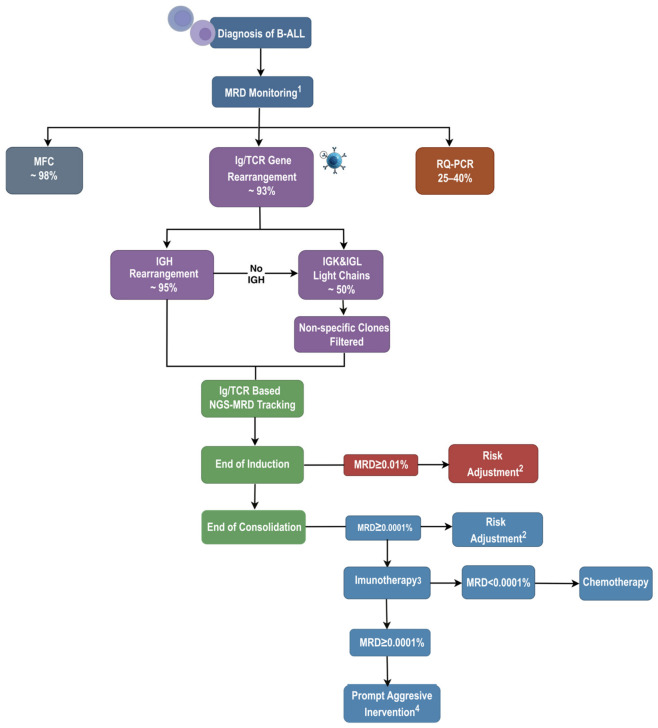
Immunoglobulin (Ig)/T-cell receptor (TCR) gene rearrangement detection and MRD-directed treatment pathway for B-cell acute lymphoblastic leukemia. ^1^ Current MRD monitoring approaches and detection rates in B-ALL [19,31,32]. ^2^ For standard or intermediate risk patients, we recommend reclassification to the high risk stratum, whereas high-risk patients may continue on monitoring. ^3^ We recommend blinatumomab, given its ability to further induce NGS-MRD clearance [33]. ^4^ Consideration of other immunotherapies, CAR T-cell therapy or HSCT.

The superior sensitivity and specificity of NGS-MRD have been validated in numerous retrospective and prospective clinical studies, establishing its clinical utility in defining therapeutic thresholds, refining prognostic stratification, monitoring treatment response after novel therapies and guiding treatment decisions. Key information for B-ALL is summarized in Figure 2. NGS-MRD has emerged as a cornerstone of personalized pediatric ALL management, addressing critical limitations of conventional MRD assays.

### 3.1. Superior Sensitivity and Specificity for MRD Detection

The core clinical advantage of NGS-MRD is its ability to detect ultra-low-level MRD that is undetectable by MFC and PCR, with consistent validation across pediatric ALL cohorts. Mai et al. [34] reported that NGS achieved a significantly higher MRD positive rate than conventional assays in both B-ALL and T-ALL. In B-ALL, NGS-MRD showed a markedly higher positive rate (57.5% vs. 26.9% for MFC and 52.1% vs. 18.8% for RQ-PCR), and this difference was even more pronounced in T-ALL (80% vs. 46.7%). Moreover, NGS-MRD exhibited strong quantitative correlations with MFC (r = 0.708) and RQ-PCR (r = 0.618), and could sensitively detect low-level leukemic clones that were missed by conventional methods. While this study was a retrospective, single-center analysis, to provide a higher level of evidence, a prospective clinical trial by Paolino et al. [35] demonstrated that among patients classified as MRD-positive (≥10^−4^) after induction, 43% of B-ALL cases and 75% of T-ALL cases were identified solely by NGS, with the majority of these discrepant cases having MRD levels at or just above the MFC detection limit of 10^−4^. Furthermore, NGS detected MRD in the range of 10^−6^ to <10^−4^ in 160 patients (50% of the cohort) who were MFC-negative at the post-induction timepoint, underscoring its superior sensitivity in the low-level MRD window. Hwang et al. [36] further confirmed that NGS detected 39.6% of MRD-positive pediatric B-ALL cases, compared with only 23.7% by MFC, and identified low-level MRD in 18% of MFC-negative cases. Compared with Allele-Specific Oligonucleotide Real-Time Quantitative (ASO)-RQPCR, NGS-MRD has a wider quantitative range and can detect ultra-low-level MRD that is undetectable with ASO-RQPCR, enabling early relapse warning (1–5 months in advance) [37]. This superior sensitivity is clinically meaningful, as ultra-low-level MRD is a strong predictor of subsequent clinical relapse—an outcome that conventional assays fail to anticipate.

### 3.2. Defining Therapeutic Thresholds and Refining Prognostic Stratification

NGS-MRD has provided a reliable molecular standard for defining MRD therapeutic thresholds and refining prognostic stratification in pediatric ALL, enabling risk-adapted treatment adjustments that balance efficacy and toxicity. Clinical studies have established disease stage-specific NGS-MRD thresholds with significant prognostic differentiation value. Patients with NGS-MRD < 0.01% at EOI or <0.0001% at EOC achieve 3-year event-free survival (EFS) ≥ 95% [19]. An independent study further confirms that normalized EOI MRD < 0.01% is a robust prognostic indicator (3-year EFS: 100% vs. 60.9% ± 10.2%, 3-year OS: 100% vs. 78.3% ± 8.6%) [38]. European studies [39] have shown that NGS-MRD revised 19% of patients into a lower-risk group by definitively excluding false-positive results from conventional qPCR, and reallocated 5% of patients to a higher-risk group by identifying true MRD positivity that qPCR failed to accurately capture, reducing unnecessary chemotherapy toxicity in overclassified low-risk patients while ensuring intensive therapy for underclassified high-risk patients.

For specialized therapies such as CAR-T cell therapy and hematopoietic stem cell transplantation (HSCT), the prognostic threshold for NGS-MRD is simplified to any detectable level of residual clonal Ig/TCR signals. Detectable BM NGS-MRD at day 28/30 and 3 months post-CAR-T cell therapy is associated with a significantly increased relapse risk, and any detectable NGS-MRD before/after HSCT has a profound negative impact on OS and relapse-free survival (RFS) [23,27,40,41]. These thresholds have been integrated into clinical practice to guide post-therapy management decisions.

### 3.3. Molecular Subtype-Specific Utility of NGS-MRD

The clinical utility of NGS-MRD varies across pediatric ALL molecular subtypes, reflecting distinct molecular biological characteristics of each subtype that influence the performance of MRD assays. NGS-MRD exhibits superior prognostic stratification ability to qPCR in the *ETV6::RUNX1* subtype [42,43]: *ETV6::RUNX1*-positive ALL has a higher number of clonal Ig/TCR rearrangements in diagnostic samples, leading to more reliable NGS-MRD detection. In contrast, NGS-MRD and qPCR have equivalent prognostic value in *TCF3::PBX1*-positive ALL, with an extremely high correlation (R^2^ = 0.9157) and consistency (95.8%) in MRD detection—a finding attributed to the unique molecular biology of this subtype, which lacks systematic deviations between the two assays.

For Philadelphia chromosome-positive (Ph+) ALL, a high-risk pediatric ALL subtype, NGS-detected MRD levels ≥ 10^−2^ at EOI were associated with a 5-year EFS of only 33 ± 8%. Meanwhile, MRD levels ≥ 10^−3^ at EOC correlated with a 5-year EFS of 36 ± 9% and OS of 53 ± 9%, serving as a molecular basis for risk stratification and treatment guidance in the typical Ph+ ALL subgroup [43]. A study of 81 Ph+ ALL patients found that ~20% of patients had *BCR::ABL1* positive but NGS-MRD-negative CML-like biological characteristics, with a 5-year RFS rate of 84% and OS rate of 94% (only 1 relapse in 20 patients with median follow-up >5 years) [44]. This key finding confirms that NGS-MRD more accurately assesses the true residual leukemic burden in CML-like Ph+ ALL, eliminating PCR false positives and avoiding unnecessary intensive therapy. Nevertheless, these findings strictly demand rigorous prospective validation in international, multicenter clinical trials to confirm whether de-escalating therapy based solely on NGS-MRD negativity is unequivocally safe in this high-risk subgroup.

### 3.4. Monitoring Treatment Response After CAR-T Cell Therapy and HSCT

CAR-T cell therapy and HSCT represent two curative treatment options for relapsed/refractory (R/R) pediatric ALL. NGS-MRD functions as a vital molecular biomarker to assess treatment response and forecast relapse following these therapies, resolving the major shortcomings of conventional MRD detection methods.

In CAR-T cell therapy (CD19-directed), NGS-MRD provides robust prognostic information [27,40]: detectable NGS-MRD at day 28 post-tisagenlecleucel therapy is independently associated with relapse risk (hazard ratio [HR] = 4.87), and this HR increases to 12 at 3 months. Any detectable NGS-MRD at months 3–6 indicated dismal outcomes. The median interval from detectable NGS-MRD to clinical relapse is 168 days, and this interval shortens to 70 days when the threshold is set at 10^−6^, compared with only 52 days for MFC-MRD. A unique molecular advantage of NGS-MRD is that clonal Ig/TCR rearrangements remain stable even after CD19 expression loss or lineage switch (a common mechanism of CAR-T resistance), enabling continuous MRD monitoring in CAR-T-refractory patients.

In HSCT, NGS-MRD is a strong predictor of post-transplant outcomes: the 1-year relapse free survival (RFS) rates of NGS-MRD-positive and -negative pediatric ALL patients are 40% and 96%, respectively, and the 3-year OS rates are 33.3% and 94.4% [45]. Notably, NGS-MRD verifies that 30–40% of “positive, non-quantitative” RQ-PCR results post-HSCT are false positives, caused by non-specific amplification of normal hematopoietic cell sequences that are highly homologous to leukemic clone sequences [46]. This finding is clinically transformative, as it spares unnecessary preemptive therapy for patients with RQ-PCR false positives without increasing relapse risk.

### 3.5. NGS-MRD-Guided Clinical Treatment Adjustments

Beyond providing precise prognostic stratification, NGS-MRD monitoring has increasingly become the definitive molecular tool for guiding therapeutic modifications and personalized interventions in pediatric ALL. In frontline and early consolidation settings, the persistent presence of ultra-low-level MRD detected exclusively by NGS identifies patients at an elevated risk of impending relapse, thereby justifying the early escalation of therapy. Specifically, the introduction of targeted immunotherapies, such as the bispecific T-cell engager blinatumomab, has demonstrated remarkable efficacy in eradicating persistent residual leukemic clones that are detectable by NGS. In patients with MFC-negative but NGS-positive MRD, a single course of blinatumomab achieved an NGS-MRD clearance rate of 68% (13/19) [33]. Another study confirmed that introducing blinatumomab consolidation for patients who remain NGS-MRD positive can rapidly achieve complete molecular clearance down to the 10^−6^ threshold, enabling a safe and effective bridge to long-term maintenance or transplantation [47].

Furthermore, in the context of salvage therapies like HSCT and CAR-T cell therapy, longitudinal NGS-MRD monitoring acts as an essential trigger for preemptive clinical interventions. Upon the detection of emergent or persistent NGS-MRD following transplantation, clinicians can promptly initiate salvage strategies, including the rapid tapering of immunosuppressive agents, administration of donor lymphocyte infusions, or early re-introduction of blinatumomab to avert overt hematological relapse [23]. Similarly, in patients receiving CD19-directed CAR-T cell therapy, the re-emergence of clonal Ig/TCR sequences captured by NGS provides a crucial early warning signal months before morphological relapse, allowing for timely therapeutic adjustments such as the infusion of alternative CAR-T constructs or immediate consolidation with HSCT [27]. Thus, the integration of NGS-MRD into standard clinical practice represents a critical paradigm shift from passive prognostic monitoring to dynamic, molecularly guided treatment adjustments.

## 4. Current Molecular and Clinical Challenges of NGS-MRD in Pediatric ALL

Despite its significant clinical advances, the widespread translation and standardization of NGS-MRD in pediatric ALL are hindered by a series of unresolved molecular, technical, regulatory and clinical challenges—these represent the key current issues in the field, and addressing them is essential for the routine clinical use of NGS-MRD.

### 4.1. Lack of Standardized Molecular Criteria for Clone Identification

A major unresolved challenge is the absence of internationally standardized molecular criteria for defining clonal Ig/TCR rearrangements and distinguishing leukemic clones from normal lymphocyte clones. There is ongoing controversy regarding the threshold for clonal sequence determination and the molecular features that differentiate leukemic from non-specific normal clones. This lack of standardization leads to inter-laboratory variability and severely affects the inter-laboratory reproducibility in NGS-MRD results, a critical barrier to its widespread clinical application.

### 4.2. Unclarified Clinical Implications of Ultra-Low-Level MRD

NGS-MRD can detect ultra-low-level MRD at 10^−6^–10^−7^, but the clinical implications of this ultra-low-level disease remain unclarified. It is unknown whether ultra-low-level MRD represents persistent leukemic blasts with relapse potential or harmless residual clonal fragments with no clinical significance, and there are no established clinical intervention indications for this subset of patients.

### 4.3. Absence of a Unified International Quality Control System

The EuroClonality-NGS consortium has proposed a standardized NGS-MRD workflow, but there is still no unified international quality control (QC) system for NGS-MRD detection in pediatric ALL. QC systems are essential for ensuring the reproducibility and reliability of NGS-MRD results across different laboratories and platforms, but current QC efforts are fragmented and limited to regional consortia. The development of a global QC system with standardized reference materials and proficiency testing is a critical unmet need.

### 4.4. Barriers to Widespread Accessibility and Variable Bioinformatics Pipelines

NGS-MRD is associated with high detection costs and stringent requirements for experimental and bioinformatics analytical platforms, which limit its accessibility in primary and resource-limited medical institutions. The bioinformatics analysis of NGS-MRD data requires specialized expertise in Ig/TCR gene rearrangement analysis and clonal identification, which is lacking in many clinical laboratories. Furthermore, the bioinformatics pipelines utilized for data filtering, error correction, and sequence alignment vary significantly across institutions. This lack of standardized bioinformatics pipelines contributes heavily to inconsistent data interpretation and poses a major challenge for multi-center data harmonization. These practical barriers prevent the equitable distribution of NGS-MRD technology, a key challenge for its global clinical translation.

### 4.5. Regulatory Issues and Translation to Population-Based Management

Incorporating NGS for MRD into regular clinical use is further complicated by existing regulatory issues. Obtaining standardized in vitro diagnostic approval for these complex, multi-gene assays and establishing sustainable reimbursement models remain significant barriers to translation to practice. Acknowledging these hurdles is essential to provide a more balanced opinion on the field’s current status. To successfully fit NGS for MRD into the population-based management of pediatric ALL, collaborative efforts among researchers, clinicians, and regulatory bodies are required to establish clear clinical guidelines, standardized reporting structures, and regulatory consensus.

## 5. Conclusions and Future Perspectives

To address the current molecular and clinical challenges and realize the full potential of NGS-MRD in pediatric ALL, future research and clinical translation efforts should focus on five core directions:

• Construct subtype-specific molecular databases: Establish an NGS-MRD database for pediatric ALL based on multicenter studies, to define molecular criteria for clone identification and normal/leukemic clone differentiation, and formulate regional and international standard operating procedures (SOPs) and QC systems to ensure inter-laboratory reproducibility.
• Optimize bioinformatics algorithms: Develop advanced bioinformatics algorithms to improve the efficiency and accuracy of identifying leukemia-specific Ig/TCR clones, and reduce the false positive rate caused by non-specific normal clones.
• Conduct prospective clinical studies: Design large-scale prospective studies to clarify the clinical implications of ultra-low-level MRD (10^−6^–10^−7^) and define evidence-based clinical intervention indications for this subset of patients.
• Navigate regulatory issues and reduce detection costs: Promote technological process optimization for NGS-MRD, actively address regulatory hurdles for clinical approval, and develop simplified, low-cost NGS platforms to improve its accessibility in primary and resource-limited institutions.
• Develop multi-marker prognostic models: Integrate NGS-MRD with other molecular biomarkers (e.g., fusion genes, somatic mutations, epigenetic modifications) to establish a comprehensive multi-marker prognostic evaluation model, enabling more precise risk stratification and personalized treatment of pediatric ALL.

In addition, the integration of artificial intelligence (AI) and machine learning algorithms provides a transformative solution for NGS-MRD data analysis. Training deep neural network models to capture the complex, high-dimensional features of Ig/TCR rearrangements supports the automated and robust identification of leukemia-specific clones and distinguishes them from non-specific background interference. Preliminary studies have verified that AI-based clonal screening models can achieve an accuracy rate of over 95% in differentiating benign rearrangements from malignant ones [48]. In the future, constructing a universal algorithmic framework by aggregating multi-center and multi-platform data will effectively resolve key bottlenecks, which will further accelerate the standardization and intelligent upgrading of NGS-MRD technology.

In summary, NGS-MRD is a powerful molecular tool for MRD detection in pediatric ALL, and addressing its current challenges will further advance its clinical translation and standardization—ultimately improving the curative efficacy and long-term outcomes of pediatric ALL patients via precision medicine.

## Figures and Tables

**Table 1:** Comparison of different minimal residual disease (MRD) detection methods.

Detection Method	Sample Type	Scope of Application	Sensitivity	Advantages	Limitations
MFC	Live cells	Applicable to >90% of children with acute leukemia, especially those lacking suitable genetic markers	0.01%	Fast, wide coverage, relatively low cost	Susceptible to interference from increased benign precursor B cells during bone marrow recovery; antigen drift and phenotypic conversion may lead to false negatives
PCR (Ig/TCR gene rearrangement)	DNA	90~95% of ALL	0.01%	High sensitivity, strong specificity, high degree of standardization	Time-consuming and complex operation; requires diagnostic samples; may miss minor clones at diagnosis
NGS	DNA	Mainly applicable to ALL	0.0001%	Extremely high sensitivity, can detect multiple Ig/TCR clones simultaneously	High cost, long cycle, requires diagnostic samples; difficult to confirm whether clones are leukemia-specific

## Data Availability

No new data were created or analyzed in this study. Data sharing is not applicable to this article.
